# Radotinib attenuates TGFβ -mediated pulmonary fibrosis in vitro and in vivo: exploring the potential of drug repurposing

**DOI:** 10.1186/s40360-022-00634-x

**Published:** 2022-12-15

**Authors:** Suji Baek, Seung Hae Kwon, Joo Yeong Jeon, Gong Yeal Lee, Hyun Soo Ju, Hyo Jung Yun, Dae Jin Cho, Kang Pa Lee, Myung Hee Nam

**Affiliations:** 1 Research and Development Center, UMUST R&D Corporation, 84, Madeul-ro 13-gil, Dobong-gu, 01411 Seoul, Republic of Korea; 2 Seoul Center, Korean Basic Science Institute, 02841 Seoul, Republic of Korea; 3 Il Yang Pharm Co.,Ltd, 37, Hagal-ro 136 Beon-gil, Giheung-gu, 17096 Yongin-si, Gyeonggi-do Republic of Korea

**Keywords:** Radotinib, Tyrosine kinase, Lung fibrosis, Extracellular signal-regulated kinase, TGF-β1

## Abstract

**Background:**

Tyrosine kinase (TK) plays a crucial role in the pathogenesis of idiopathic pulmonary fibrosis. Here, we aimed to investigate whether radotinib (Rb) could inhibit pulmonary fibrosis by inhibiting TK in vitro and in vivo.

**Methods:**

The antifibrotic effects of Rb in transforming growth factor-β (TGF-β)1-stimulated A549 cells were determined using real-time polymerase chain reaction, western blotting, and immunocytochemistry assays. Rb inhibition of bleomycin-induced lung fibrosis in Sprague Dawley (SD) rats was determined by histopathological and​ immunohistochemical analyses. Rb-interfering metabolites were analyzed using LC-MS/MS.

**Results:**

Rb concentrations of up to 1000 nM did not affect the viability of A549 cells, but Rb (30 nM) significantly reduced expression of TGF-β1 (10 ng/mL)-induced ECM factors, such as Snail, Twist, and F-actin. Rb also regulated TGF-β1-overexpressed signal cascades, such as fibronectin and α-smooth muscle actin. Furthermore, Rb attenuated the phosphorylation of Smad2 and phosphorylation of kinases, such as, extracellular signal-regulated kinase, and protein kinase B. In the inhibitory test against bleomycin (5 mg/kg)-induced lung fibrosis, the Rb (30 mg/kg/daily)-treated group showed a half-pulmonary fibrosis region compared to the positive control group. In addition, Rb significantly reduced collagen type I and fibronectin expression in the bleomycin-induced fibrotic region of SD rats. Further, the identified metabolite pantothenic acid was not altered by Rb.

**Conclusion:**

Taken together, these results indicate that Rb inhibits TGF-β1-induced pulmonary fibrosis both in vitro and in vivo. These findings suggest that Rb may be an effective treatment for pulmonary fibrosis-related disorders and idiopathic pulmonary fibrosis.

**Supplementary Information:**

The online version contains supplementary material available at 10.1186/s40360-022-00634-x.

## Background

Diffuse parenchymal lung disease (DPLD) is an intractable respiratory disease that is difficult to treat and is characterized by a change in the shape of the lungs and a decrease in oxygen supply to the pulmonary blood vessels [1, 2]. Cases where the cause of DPLD is unknown are classified as idiopathic pulmonary fibrosis (IPF) [3]. IPF, which has the worst prognosis among DPLD, has an incidence of 10 per 100,000 people, and an average survival of only 3 to 5 years [4, 5]. Current knowledge in the field suggests that the increased incidence of IPF is related to the long-term effects of COVID-19 infection and severe respiratory syndrome [6].

Fibrosis is caused by the accumulation of extracellular matrix (collagen and fibronectin) in the lung tissue damage during the inflammation healing process, causing lung dysfunction [7]. However, the etiological molecular mechanisms underlying IPF have not been clearly elucidated [8]. Recent studies and clinical trials have suggested that transforming growth factor-β (TGF-β)-1 and tyrosine kinase (TK) signaling are both potential IPF treatments [9, 10]. TKs, which includes platelet-derived growth factor receptor (PDGFR)-α, PDGFR-β, vascular endothelial growth factor receptor (VEGFR), and epidermal growth factor, are membrane receptors that activate intracellular signaling pathways when growth factors bind to their extracellular domains [11, 12]. TK also induces fibrosis by activating Smad, mitogen-activated protein kinase (MAPK), and protein kinase B (AKT) [13]. Therefore, subsignal modulation of TK could be a key strategy in IPF treatment.

Nintedanib and pirfenidone, both FDA-approved TK targeting-drugs, are the standard treatment for IPF, which have been reported to reduce lung function decline by 50% [14]. Although these drugs prevent pulmonary fibrosis by inhibiting TGF-β and TNF-α, there is a continuous demand for research for new therapeutic agents for IPF. Radotinib (Rb) is a TK that inhibits breakpoint cluster region protein-v-abelson murine leukemia viral oncogene homolog 1 and PDGFR and has been approved by the Korea Ministry of Food and Drug Safety (republic of Korea) approval and is being used as a treatment for acute leukemia [15]. However, studies on the efficacy of Rb against IPF have not yet been conducted. Therefore, this study not only confirmed the potential of Rb for the prevention and treatment of pulmonary fibrosis, but also confirmed the possibility of drug re-purposing by changing the use of an approved drug. In this study, we explored the anti-fibrosis effect of Rb in TGF-β1-stimulated A549 cells and in a bleomycin-induced rat model to provide basic data for the development of therapeutic agents through the significant inhibition of IPF.

## Methods

### Cell culture of A549 cells

A549 cells were purchased from the Korean Cell Line Bank (Seoul, Republic of Korea). Cells were cultured in Roswell Park Memorial Institute Medium 1640 media containing 10% fetal bovine serum (FBS) and 1% penicillin–streptomycin at 37 ± 2 °C and 5% CO_2_.

### Cell viability assay

A549 cells were seeded in 96-well plates (1 × 10^4^ cells/well) and treated with various concentrations (3, 10, 30, 100, 300, and 1000 nM) of Rb or pantothenic acid (PA; 1, 3, 10, 30, 100, 300 and 1000 µM) for 24 h. The cells were incubated with 4-[3-(4-Iodophenyl)-2-(4-nitrophenyl)-2 H-5-tetrazolio]-1,3-benzene Disulfonate-1 reagent for 2 h at 37 ± 2 °C, and cell viability was measured at 450 nm using an iMARK microplate reader (Bio-Rad, Hercules, CA, USA).

### Analysis of mRNA expression using the real-time PCR

The mRNA expression analysis was performed as previously described [16]. Total RNA was isolated from A549 cells using TRIzol reagent according to the manufacturer’s instructions. cDNA was synthesized from 1 µg of total RNA using a Superscript III First-Strand Synthesis Kit. The mRNA expression of *Snail* and *Twist* in A549 cells was measured using quantitative real-time polymerase chain reaction (PCR) with SYBR Green PCR mix (Bioneer, Daejeon, Republic of Korea). The primer sequences for *Snail* were 5- TGC ACC ACC AAC TGC TTA GC-3 (forward) and 5-GGC ATG GAC TGT GGT CAT GAG-3 (reverse); *Twist*, 5 -GGA GTC CGC AGT CTT ACG AG-3 (forward) and 5- TCT GGA GGA CCT GGT AGA GG -3′ (reverse); and those for *β-actin* were 5-GTC TTC CCC TCC ATC GT -3’ (forward) and 5-CGT CCC CAC ATG GAA T-3 (reverse). The cycling conditions were as follows: initial denaturation at 95 °C for 10 min, followed by 40 cycles of denaturation at 95 °C for 10 s, annealing at 60 °C for 30 s, and extension at 72 °C for 30 s. The relative mRNA levels were calculated using the 2^−ΔΔCt^ method and normalized to those of β-actin.

### ***In vitro*** immunocytochemistry

A549 cells (5 × 10^3^ cells/mL) were seeded in a 35-mm confocal dishes and treated with or without TGF-β1 or Rb for 24 h. A549 cells were fixed in 4% paraformaldehyde for 10 min at room temperature (RT), and permeabilized in 0.1% Triton X-100 for 10 min at RT. Samples were blocked in 5% bovine serum albumin (BSA) for 1 h, and then incubated with the E-cadherin, alpha smooth muscle actin (α-SMA) and F-actin probe (1:1,000), while nuclei were stained with DAPI. Images were obtained at 200 × magnification using a fluorescence microscope (Laser scanning microscopes 780, Zeiss, Oberkochen, Germany).

### Immunoblotting assay

Western blotting was performed as previously described [17]. The A569 cells were seeded (1 × 10^6^ cells/mL) in a 100-mm dish. Cells were incubated in FBS-free DMEM for 24 h and treated with or without TGF-β1 (10 ng/mL) and Rb (30 nM) for 30 min. After cells were lysed, proteins were separated by 12% polyacrylamide gel electrophoresis and transferred onto polyvinylidene fluoride membranes at 4 °C for 2 h. The membranes were blocked with 5% BSA for 1 h at 25 °C and incubated with specific antibodies, such as ERK1/2, phosphorylated ERK1/2 (p-ERK1/2), p38, P-p38, AKT, p-AKT, Smad2, p-Smad2, α-SMA, or β-actin (each antibody at a dilution of 1:500), for 18 h at 4 °C. After washing with Tris-buffered saline-Tween 20, the membranes were incubated with a 1:500 dilution of secondary antibody (conjugated horseradish peroxidase) for 1 h. Protein levels were detected by chemiluminescence and analyzed using ImageJ software.

### Animal care and Rb treatment in bleomycin-induced lung fibrosis mice model

Six-week-old male Sprague Dawley (SD) rats (Orient Bio, Kyunggi-do, Republic of Korea) were used for experiments after acclimation for 1 week. The experimental animals were housed in standard cages in a breeding room maintained at a constant temperature of 25 ± 2 °C, humidity 55 ± 5%, and a 12-h light/dark cycle, and were provided a standard feed (5L79 formula) containing 18% protein, 0.85% calcium, and 0.62% phosphorus. Application of bleomycin-induced lung fibrosis rat model was performed as previously described [18]. The SD rats (n = *15*) were randomly divided into three groups: untreated (UN, injected with 50 µL of saline/daily for 21 days), bleomycin treated (Bl, co-treated with 5 mg/kg of bleomycin and 0.5% carboxymethyl cellulose (CMC) for 21 days), and the bleomycin and Rb treated (Bl + Rb, co-treated with 5 mg/kg of bleomycin and 30 mg/kg/day of Rb in 0.5% CMC for 21 days) group. Lung tissue and blood were collected from sacrificed SD rats.

### Histochemistry and immunochemistry

Histochemistry and immunohistochemistry were performed as previously described [19]. Lung tissues were fixed in 4% formalin, and sections were embedded in paraffin, and serially sectioned into 5-µm slices. The prepared sections were cleared with xylene and hydrated using an ethanol gradient (70, 80, and 90%). Hematoxylin and eosin staining, or the Masson’s trichrome (M/T) staining were performed to examine the histological changes in the lung tissues. The stained specimens were analyzed under a microscope (Axioscan 7; Zeiss). The damaged areas in the lung tissues were measured using ImageJ software. Some sections were incubated with anti-collagen type I (COLI) and anti-fibronectin primary antibodies overnight at 4 °C, and then incubated with secondary antibodies. Tissues were treated with ABC reagent (Vector Laboratories, Burlingame, CA, USA) and DAB solution (Vector Laboratories). Photographs were obtained using K1-fluo (Nanoscope Systems, Daejeon, Republic of Korea). The intensities of collagen type I (Col I) and fibronectin were measured using ImageJ software.

### Analysis of metabolites in mice serum using LC/Mass

Serum isolation and metabolites analysis were performed as previously described, with slight modifications [20]. Briefly, serum samples from rats were collected after 4 weeks. Blood samples from each group were collected and placed on ice for 30 min, followed by centrifugation at 3,000 rpm for 10 min at 4 °C. The prepared serum samples were stored at − 80 °C. Each serum sample (20 µL) was prepared by adding 80 µL of 80% methanol and then vortexing for 1 min. The mixture was incubated at 4 °C for 1 h. The samples were then centrifuged at 14,000 rpm and 4 °C for 20 min. The supernatant was injected into an ultra-performance liquid chromatography/quadrupole time-of-flight mass spectrometer (UPLCQ/TOF-MS; Waters Corporation, Milford, MA, USA). The metabolites in the serum were separated using an Acquity UPLC BEH C18 column (2.1 × 100 mm, 1.7 μm particle size: Waters Corporation). The column temperature was maintained at 40 °C. The mobile phases consisted of 0.1% formic acid in water (A) and 0.1% formic acid in acetonitrile (B). The flow rate was set to 0.4 mL/min. The samples were eluted under the following conditions: initial 0% B to 5% at 1 min, to 50% A at 2.1 min, to 95% B at 9 min, to 100% B at 10.5 min, and 0% B at 1 min, followed by equilibration for an additional 2 min. Mass acquisition was performed in the positive and negative electrospray ionization modes. Mass data were collected in the range of m/z 60–1400 for 11 min, with a scan time of 0.15 s. The source and desolvation temperatures were 110 and 450 °C, respectively.

### Processing and analysis of mass spectrometry data

Progenesis QI software (Waters Corporation) was used for data processing, including mass ion alignment, normalization, and peak picking. The intensities of the mass peaks for each sample were normalized according to the total ion intensity and Pareto scaled using the SIMCA-P + 12 software (Umetrics, San Jose, CA, USA).

### Statistical analysis of experimental results

All data were analyzed using GraphPad Prism 5.0 (GraphPad, Inc., San Diego, CA, USA). The results are expressed as the mean ± standard deviation of at least three independent experiments (n ≥ 3). The results were assessed using the Student’s *t*-test and one-way analysis of variance, followed by Tukey’s multiple range test. Statistical significance was set at P < 0.05.

## Results

### Rb inhibits the epithelial mesenchymal transition-related factors in TGF-β1-induced A549 cells

**Fig. 1:**
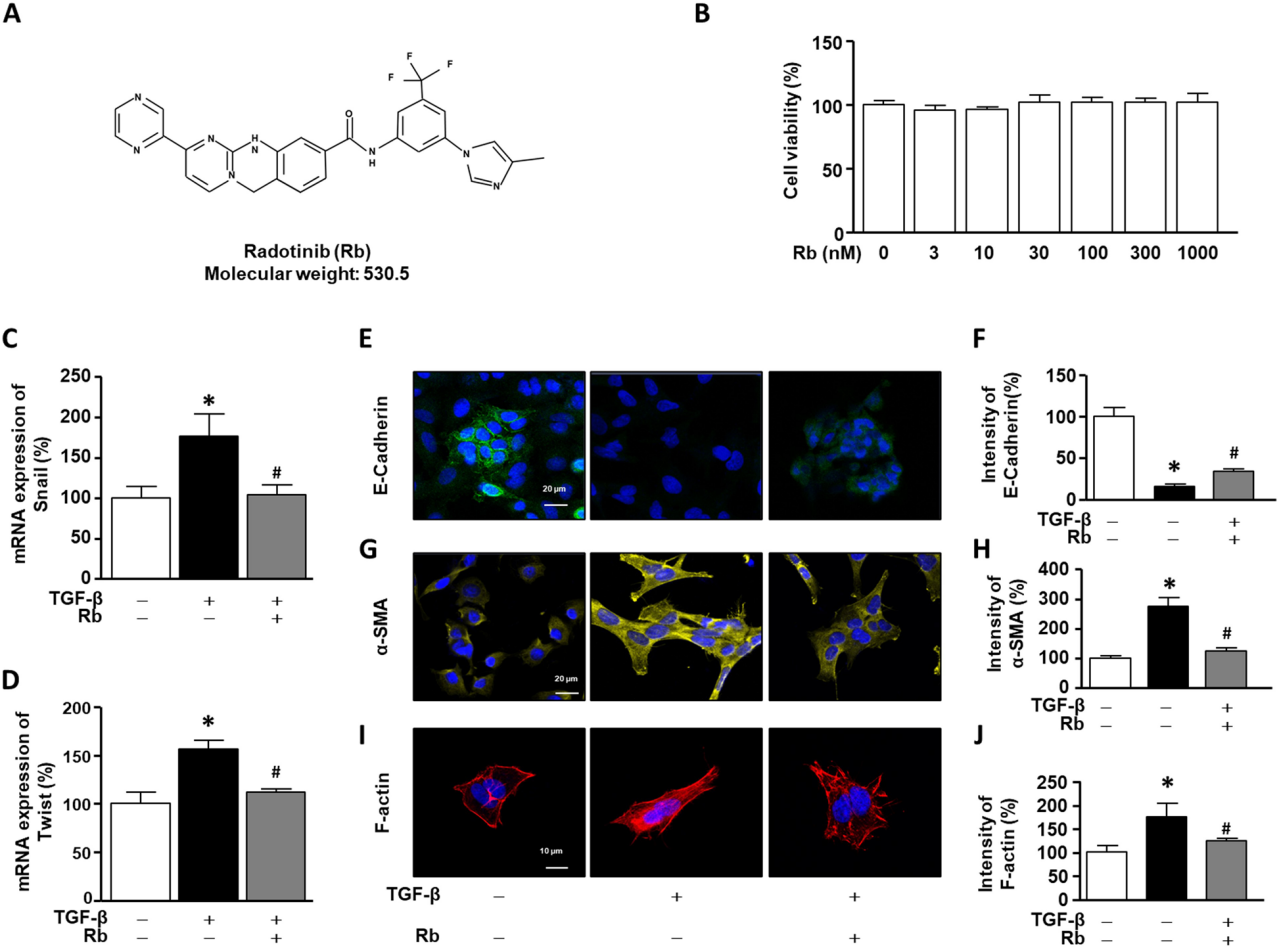
Anti-fibrosis effect of Radotinib (Rb) on TGF-β induced A549 cells. **A** Chemical structure of Rb. **B** A549 cells were incubated for 24 h, and then treated with Rb (3–1000 nM) for 24 h. Cytotoxicity was determined using a 2,3-bis[2-methyloxy-4-nitro-5-sulfophenyl]-2 H-tetrazolium-5-carboxanilide assay. **C** and **D** The cells were incubated with (+) or without (-) TGF-β1 (10 ng/mL) and Rb (30 nM) for 72 h. The mRNA expression of Snail and Twist were normalized to β-actin. (E-J) Immunocytochemistry assay with E-cadherin (Green color), α-smooth muscle actin (α-SMA, Yellow color) and F-actin (Red color) and DAPI (4′,6-diamidino-2-phenylindole). The bar graphs indicate the intensity of E-cadherin, α-SMA and F-actin, respectively. The untreated group (UN) is expressed as 100%. Data are expressed as means ± standard deviations (*n* = 3). * *p* < 0.05 vs. UN; # *p* < 0.05 vs. TGF-β1

Rb has the chemical formula C_27_H_21_F_3_N_8_O and a molecular weight of 530.50 (Fig. 1A). The cytotoxicity of Rb was determined by treating A549 cells with various concentrations of Rb (3, 10, 30, 100, 300, and 1000 nM) for 24 h. No toxicity was observed at concentrations up to 1000 nM (Fig. 1B and Fig. S1). In subsequent experiments, the optimal Rb concentration was determined to be 30 nM. To determine the effect of Rb on the TGF-β1-induced epithelial mesenchymal transition (EMT) transcription factors in A549 cells, we performed real-time PCR and immunocytochemistry assays. A549 cells were incubated in the presence or absence of TGF-β1 (10 ng/ml) and Rb (30 nM) for 24 h. In the case of mRNA expression, Rb diminished the upregulated mRNA expression of TGF-β1-stimulated targets in A549 cells, including Snail and Twist (Fig. 1C and D). In immunocytochemistry, the intensity of E-cadherin was decreased in TGF-β1-stimulated A549 cells, whereas Rb upregulated E-cadherin expression. Also, α-SMA and F-actin was increased in TGF-β1-stimulated A549 cells, whereas Rb downregulated F-actin levels (Fig. 1E-J).

### Rb reduced the phosphorylation of Smad2 and MAPK in TGF-β1-induced A459 cells

**Fig. 2:**
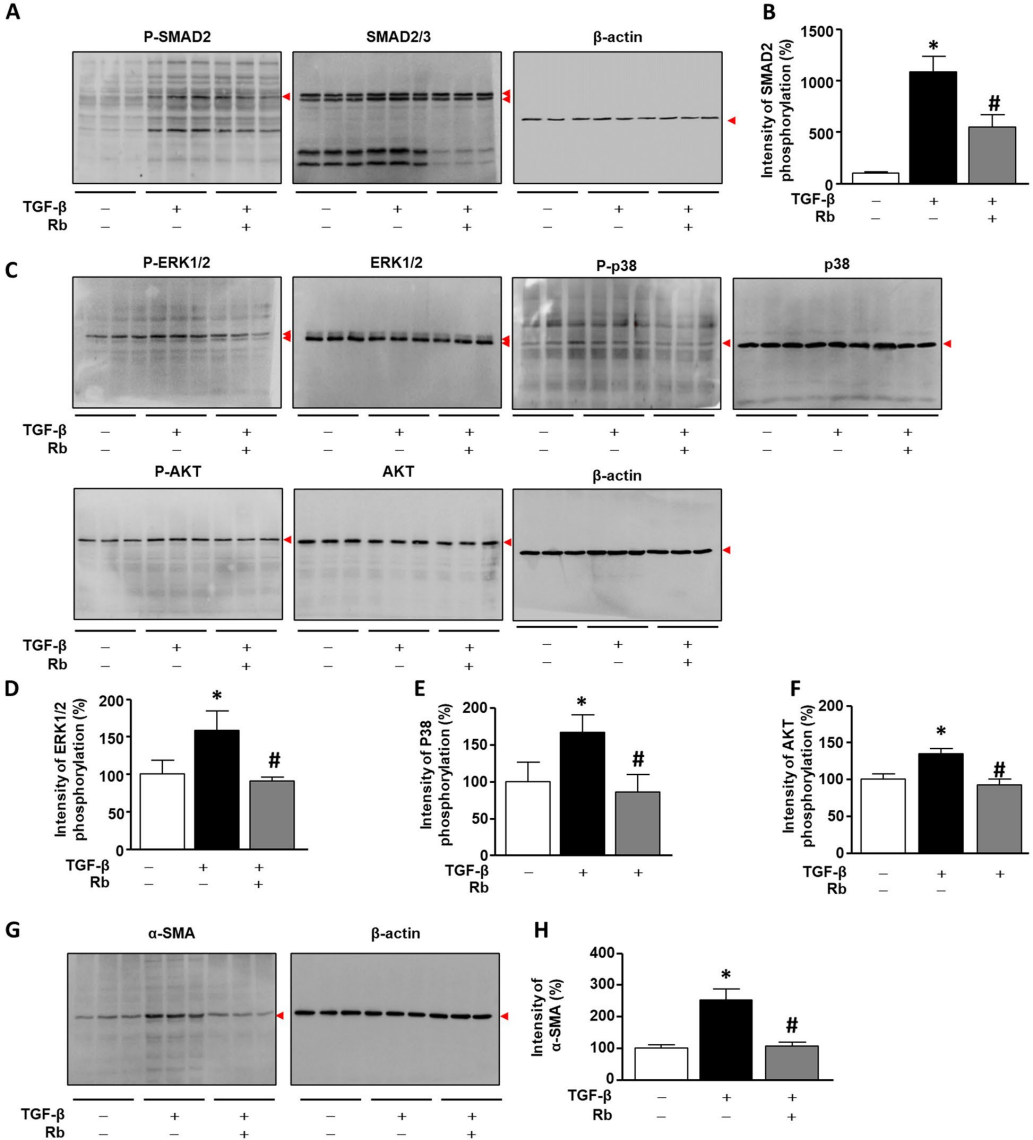
Radotinib (Rb) inhibits TGF-β1 induced tyrosine kinase signaling pathway in A549 cells. A549 cells were incubated for 24 h, and then stimulated with (+) or without (-) TGF-β1 (10 ng/mL) and Rb (30 nM) for 30 min or 72 h. (A-H) Protein expression was analyzed by western blot and incubated with specific antibodies against phosphorylated (p)-Smad2, Smad2/3, p-ERK, ERK, p-P38, P38, p-AKT, AKT, α-SMA and β-actin. The bar graphs show the intensity of western blot bands. The untreated group (UN) is expressed as 100%. Data are expressed as means ± standard deviations (*n* = 3). * *p* < 0.05 vs. UN ; # *p* < 0.05 vs. only treated with TGF-β1 group

A549 cells (1 × 10^6^ cells/well) were incubated for 24 h, and then with TGF-β1 and Rb for 30 min. Subsequently, the collected cells were lysed. To determine whether Rb can regulate TGF-β1 and tyrosine kinase signaling cascades, we performed western blotting with specific antibodies against p-Smad2, p-ERK, p-P38 and p-AKT. As shown in Fig. 2 A and B, TGF-β1 significantly increased p-Smad2 phosphorylation to 985.1 ± 140.7% compared with the untreated group, whereas Rb significantly decreased the TGF-β1-induced Smad2 phosphorylation to 536.0 ± 29.5% compared with the TGF-β1 group. In the case of ERK 1/2, TGF-β1 significantly increased ERK 1/2 phosphorylation to 59.5 ± 6.6% compared with the untreated group, whereas Rb significantly decreased the TGF-β1-induced p-ERK1/2 to 68.9 ± 19.1% compared with the TGF-β1 group (Fig. 2 C, D and Fig. S2). Similarly, Rb significantly attenuated the TGF-β1-induced phosphorylation of P38 expression up to same level as the UN group (Fig. 2 C, E and Fig. S2). In the case of AKT, 10 ng/mL of TGF-β1 significantly increased phosphorylation of AKT up to about 34.2 ± 0.5%, while Rb effectively decreased the TGF-β1-stimulated p-AKT to 42.0 ± 0.5% (Fig. 2 C, F and Fig. S2). Next, A549 cells (1 × 10^6^ cells/well) were incubated for 24 h, and then with TGF-β1 and Rb for 72 h. In the case of EMT expression, TGF-β1 significantly increased the α-SMA to 252.6 ± 33.5% compared with the untreated group. In contrast, Rb significantly decreased the TGF-β1-induced α-SMA to 105.9 ± 13.2% %, compared with the TGF-β1 group (Fig. 2G, H and Fig. S2).

### Anti-fibrosis effect of rb in bleomycin-induced lung fibrosis

**Fig. 3:**
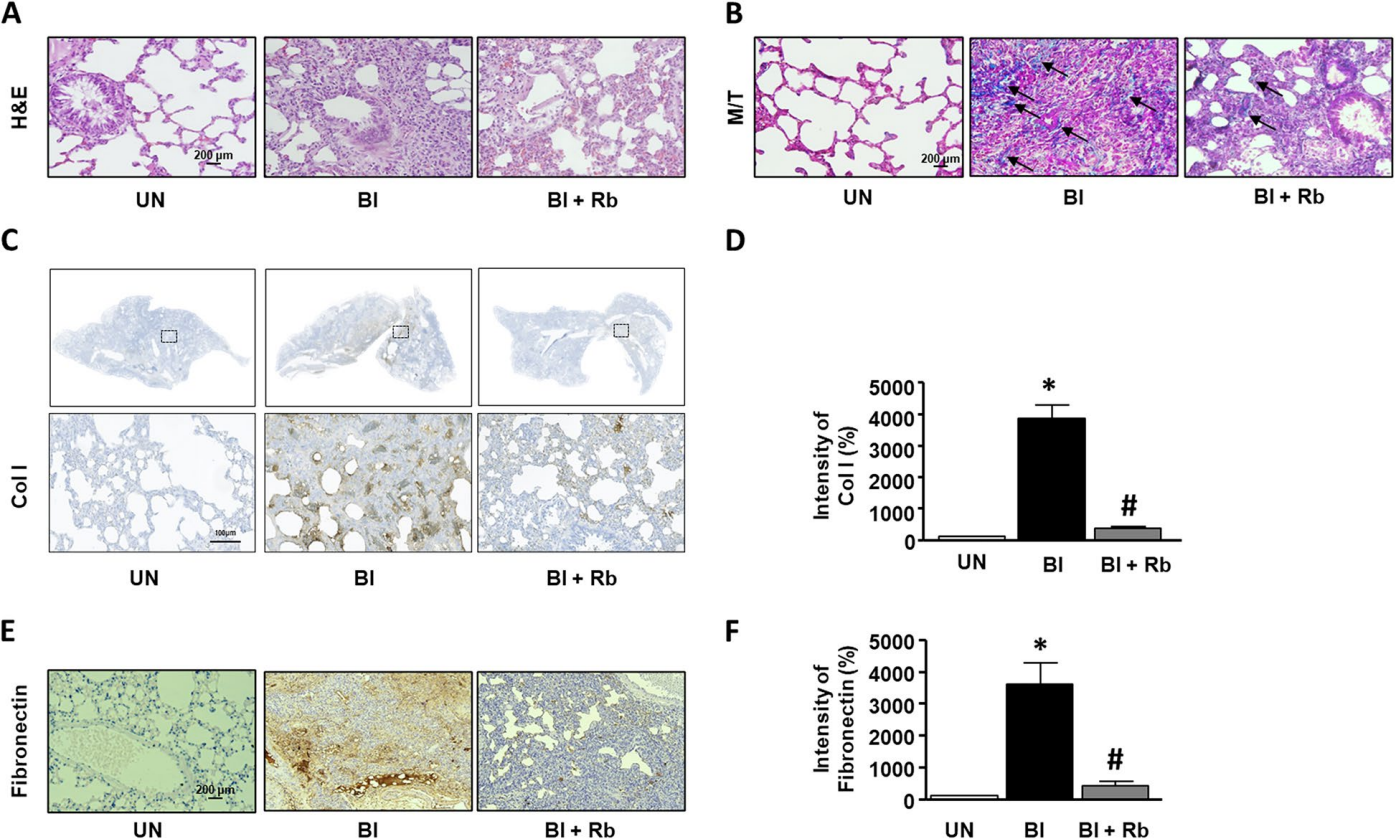
Effect of Radotinib (Rb) on lung fibrosis in bleomycin-induced Rat model. The rats were divided three groups; untreated group (UN): administered with water; Bleomyin induced group (Bl): treated with Bleomycin (5 mg/kg); bleomycin and Rb group (Bl + Rb): rats were treated with 5 mg/kg of Bleomycin and administered with Rb (30 mg/kg) daily for 21 days. Rats were sacrificed, and lung tissues were isolated. **A** Lung tissues were stained with hematoxylin and eosin. **B** Lung tissues were stained with Masson’s trichrome (M/T). The black arrows indicate the collagen accumulation. **C** and **E** The tissues were stained with specific antibodies such as anti-collagen type I (Col I) and fibronectin. Brown color indicates a positive signal. **D** and **F** The bar graphs show the brown intensity of the left photographs, respectively. The intensity of UN is expressed as 100%. Data are expressed as means ± standard deviations (*n* = 4). * *p* < 0.05 vs. UN ; # *p* < 0.05 vs. Bl

To confirm whether Rb regulates bleomycin-induced lung fibrosis in vivo, we performed histological and immunohistochemical analyses. Rats were treated with 5 mg/kg bleomycin and 30 mg/kg Rb for 21 days. As shown in Fig. 3 A and B, the lung tissues were stained with H&E and M/T. To examine lung fibrosis, the lung tissue was stained with Col I and fibronectin antibodies. The expression of Col I and fibronectin in the bleomycin-induced group significantly increased compared to that in the untreated group, while treatment with bleomycin and Rb significantly inhibited the expression of Col I and fibronectin, respectively (Fig. 3 C-F).

**Fig. 4:**
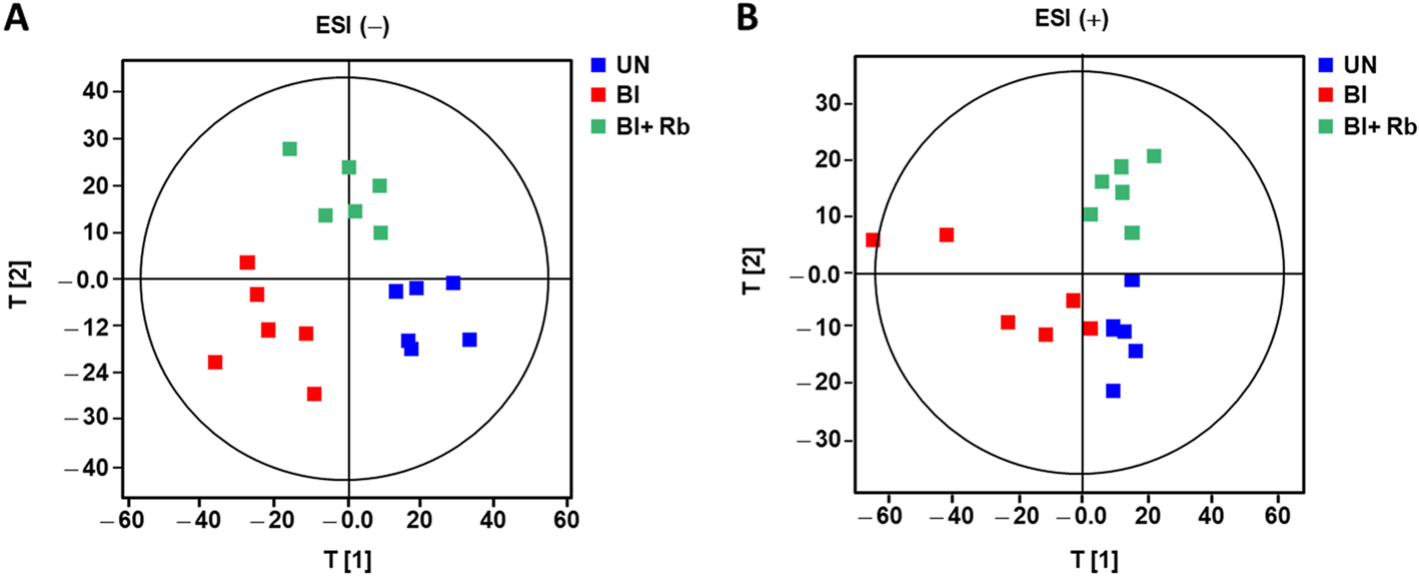
Metabolic change analysis. Partial least-squares discriminant analysis (PLS-DA) score plots of control (UN: untreated group), treated with bleomycin (Bl: 5 mg/kg of bleomycin), and treated Bl + Rb (treated with 5 mg/kg of bleomycin and administered with 30 mg/kg of radotinib) groups. PLS-DA score plots were derived from metabolite ions acquired from ESI- (**A**) and ESI+ (**B**) modes (*n* = 4)

To confirm whether serum metabolite patterns changed with IPF progression, we performed global metabolite profiling and metabolite pattern analysis to monitor IPF progression and the effect of Rb on the progression of bleomycin-induced IPF. The serum metabolite profiles of the UN, Bl, and Bl + Rb groups were analyzed using UPLC-Q/TOF MS and applied to a PLS-DA model. The PLS-DA score plot showed clear discrimination between the UN, Bl, and Bl + Rb groups, indicating that these three groups have distinct metabolite profiles. Furthermore, the Bl + Rb group was distributed between the UN and bleomycin-treated groups (electrospray ionization: ESI (-)) or was on the same side as the control groups (ESI (+)), indicating that changes in metabolic pattern due to bleomycin are ameliorated by Rb (Fig. 4). This result is in good agreement with the results that Rb inhibits TGFβ1-induced pulmonary fibrosis in vitro and in vivo (Figs. 1, 2 and 3). Among the metabolites altered by bleomycin treatment, oxotetradecanoic acid, pantothenic acid, and indole-lactic acid were identified as metabolites increased by bleomycin treatment. Some phospholipid like lysophosphatidylcholine (LysoPC) (22:6), LysoPC (20:2), lysophopshatidylethanolamine (LysoPE)(20:0) were decreased in Bl group. Heatmap analysis with metabolites ions selected as having significantly different abundance between UN and Bl group (annotated and unannotated) showed a distinction of several metabolites between Bl and Bl + Rb groups For example, indolelactic acid, oxotetradecanoic acid, LysoPC (22:6), LysoPC (20:2), which were increased or decreased by bleomycin were recovered to control level by Rb treatment. On the other hands, pantothenic acid was not changed by Rb treatment (Fig. S3).

## Discussion

In the present study, we explored the potential of IPF treatment with Rb and drug repositioning. Here, we demonstrated the anti-fibrotic effect of Rb, which is involved in the TGF-β1/Smad/MAPK/AKT pathway. Our results showed that Rb inhibited ECM production in TGF-β1-stimulated A549 cells and bleomycin-induced fibrosis in SD rats. These data were obtained by western blotting, RT-PCR, histological analysis, and immunohistochemistry assays. This result implied that the inhibitory effect of the tyrosine kinase Rb could modulate TGF-β1-induced pulmonary fibrosis.

TKs include receptor tyrosine kinases, the Src family of tyrosine kinases, and BCR-ABL. TK protein kinases modulate cellular activity by transducing intracellular signals. The cellular consequences of TK activation are complex, and strongly depend on the cell type and signal transduction pathway that is activated [21]. Key signaling pathways activated by TKs include the MAPK pathways comprising Ras–ERK, p38, and JNK (also known as stress-activated protein kinase), phosphatidylinositol 3-kinase–Akt, and Janus kinase–signal transducer of activated transcription pathways. To decrease the activity of TK, inhibitors, such as nintenadib, pirfenidone, imnatinib, and radotinib, bind to the enzyme. Nintedanib is a potent intracellular inhibitor of fibroblast growth factor receptors (FGFRs)1, 2, and 3, PDGFRα and β, and vascular endothelial growth factor receptors (VEGFRs)1, 2, and 3 [22]. It also inhibits the Src family tyrosine kinases, Lck, Lyn, and Flt-3. Lin et al. reported that nintedanib inhibits the expression of p38MAPK and ERK1/2 in TGF-β1-induced human Tenon’s fibroblast cells [23]. Rb, a TK inhibitor, inhibits activated BCR-ABL and PDGFR [24]. Similar to nintenib, Rb reduced the expression of ERK1/2, p38 and AKT in TGF-β1-induced A549 cells. Therefore, Rb may be a candidate for the treatment of IPF.

Various pulmonary fibrosis models have been extensively used to investigate IPF treatments. Among these, the most commonly used model is the bleomycin-induced rodent model. Bleomycin has been reported to induce pulmonary fibrosis progression and Col I, fibronectin, and Smad signaling. Nintedanib reduced lung inflammation and fibrosis, as demonstrated by reduced lung collagen and histology in bleomycin-treated mice, and in a mouse model of silica-induced pulmonary inflammation and fibrosis [25]. In the present study, Rb also decreased the expression of Col I and fibronectin in bleomycin-treated rats. Rb also decreased the expression of Smad signaling and EMT factors, such as E-cadherin, α-SMA, Snail, and Twist in TGF-β1-induced A549 cells. Therefore, Rb may be a candidate for IPF treatment. However, we raise the question of whether Rb can exert a therapeutic effect without side effects.

To address these questions, we explored the modulation of metabolites in the blood following treatment. Our results showed that the blood vitamin B5 concentration significantly increased in bleomycin-induced SD rats compared with the normal condition (Fig. 4 and Fig S3). Vitamins that regulate metabolism in living organisms act as catalysts for enzymatic reactions, or as coenzymes that carry chemical groups between them [26]. In particular, vitamins play a very important role in the metabolic pathways of mitochondrial respiration and energy production, which maintain essential vital activities in vivo. Vitamin B5 (pantothenic acid, PA) has been reported to inhibit the progression of fibrosis in vitro [27]. Also, Ermis et al. suggested that PA protects against pulmonary fibrosis [27]. In our results, PA also significantly reduced the increase in EMT transcriptional factor in TGF-β**1**-stimulated A549 cells (Fig. S4). If Rb could cause side effects, Rb would be expected to block fibrosis indirectly by increasing PA. However, Rb treatment did not alter PA-metabolite in bleomycin-induced fibrosis in SD rats (Fig. 4 and Fig. S3). Therefore, we suggest that Rb attenuates pulmonary fibrosis by directly blocking TKI signaling.

The pharmacological action of most compounds may be accompanied by side effects in addition to the desired target [28]. The process of developing a new drug takes more than ten years on average, involving exploration of not only drug efficacy, but also an evaluation of human safety underlying drug side effects. Therefore, recent studies have suggested an approach for repurposing or repositioning drugs [29]. This research method is involved in a new methodology of new drug development that attempts to treat both common and rare diseases after repurposing drugs of potentially risk-free compounds to overcome the shortcomings of new drug development [29]. Rb is an anticancer drug that is expected to be effective in the treatment of acute leukemia and Parkinson’s disease. In addition, pharmacological stability has been reported in the human body [30]. Therefore, we suggest that Rb is a safe and effective drug for IPF treatment.

## Conclusion

Our data showed that Rb significantly inhibited TGF-β1-induced overexpression of tyrosine kinase signaling and downstream Smad signaling, both in vitro and in rat models. These results suggest that Rb may be a therapeutic agent for IPF which acts by inhibiting pathologies of pulmonary fibrosis, such as collagen and EMT production. However, further studies require quantitative analysis using biochemical assays (hydroxyproline and sircol) of fibrosis to confirm clear evidence that Rb inhibits lung fibrosis.

## Supplementary Information

**Additional file 1.**

## Data Availability

All data generated during this study are included in this published article.

## Abbreviations

TK: tyrosine kinase
Rb: radotinib
TGF-β: transforming growth factor-β
DPLD: diffuse parenchymal lung disease
IPF: idiopathic pulmonary fibrosis
RTK: receptor tyrosine kinase
VEGFR: vascular endothelial growth factor receptor
MAPK: mitogen-activated protein kinase
JNK: c-Jun N-terminal kinase
CMC: carboxymethyl cellulose
Col I: collagen type I
PA: pantothenic acid
FGF: fibroblast growth factor receptor
ECM: extracellular matrix
EMT: epithelial mesenchymal transition
