# Supplementary material for: Radotinib attenuates TGFβ -mediated pulmonary fibrosis in vitro and in vivo: exploring the potential of drug repurposing
Source: BMC Pharmacol Toxicol. 2022 Dec 15;23:93. doi: 10.1186/s40360-022-00634-x (PMC9753032; doi:10.1186/s40360-022-00634-x)
Supplement: Supplementary file 1 — Additional file 1. [file 40360_2022_634_MOESM1_ESM.doc]

**Supplementary information**

**Radotinib attenuates TGFβ -mediated pulmonary fibrosis *in vitro* and *in vivo:* Exploring the potential of drug repurposing**

Suji Baek^1*^, Seung Hae Kwon^2*^, Joo-Yeong Jeon^2^, Gong Yeal Lee^3^, Hyun So Ju^3^, Hyo Jung Yun^3^, Dae Jin Cho^3^, Kang Pa Lee^1#^ and Myung Hee Nam,^2#^

^1^Research and Development Center, UMUST R&D Corporation, Seoul 01411, Republic of Korea

^2^Seoul Center, Korean Basic Science Institute, Seoul 02841, Republic of Korea

^3^Il Yang Pharm Co.,Ltd, 37, Hagal-ro 136 Beon-gil, Giheung-gu, Yongin-si, Gyeonggi-do, 17096, Republic of Korea .

**Figure S1.** Analysis of cytotoxicity using the Hoechst staining.

**Figure S2.** Immunoblotting of diverse protein expression in TGF-β-stimulated A549 cells.

**Figure S3.** Heatmap of metabolites ions differentially regulated among groups.

**Figure S4.** Pantothenic acid inhibits TGF-β1-induced-EMT transcription factors in A549 cells.


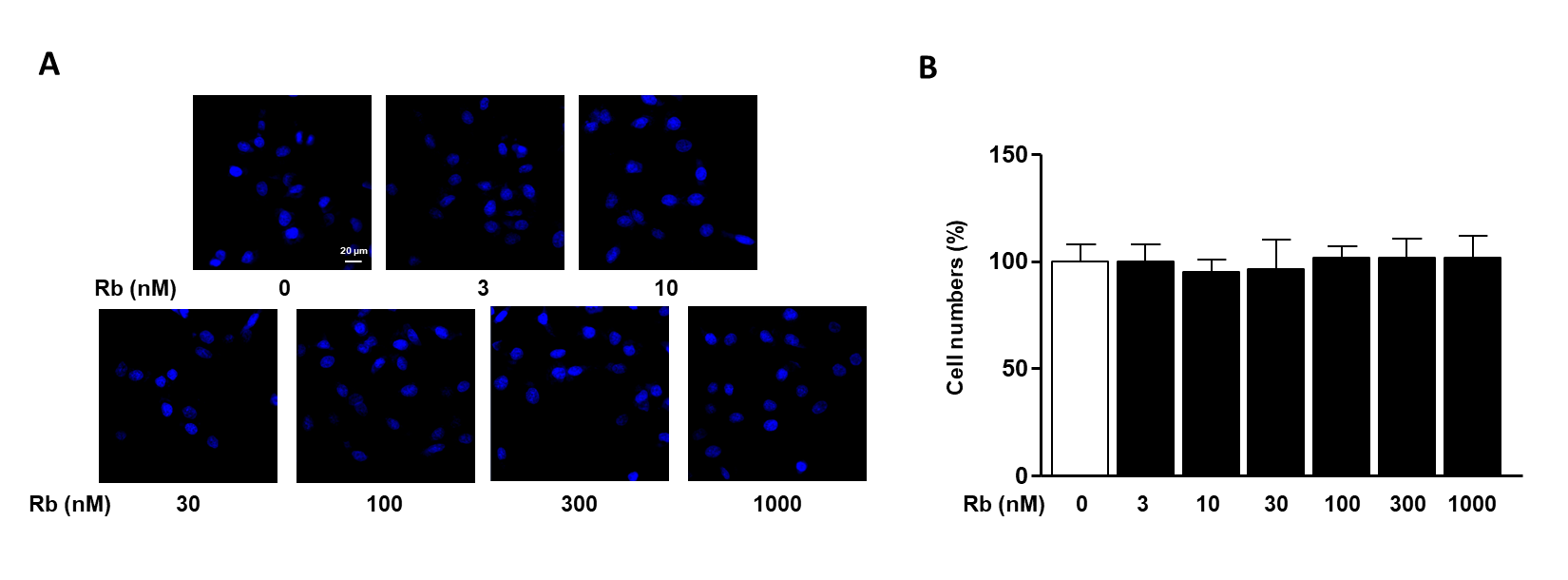


**Figure S1. Analysis of cytotoxicity using the Hoechst staining.** The A549 cells were incubated at RPMI 1640 (supplementary 10% FBS) for 24 h, and then cells were replaced at RPMI 1640 contained with radotinib (0, 3, 10, 30, 100, 300 and 1000 nM) for 24 h. The cells were stained with Hoechst 33342 for 10 min. (A) The images were obtained using the fluorescence microscopy. (B) This graph shows the quantification of cell viability using the Hoechst. Radotinib: Rb.


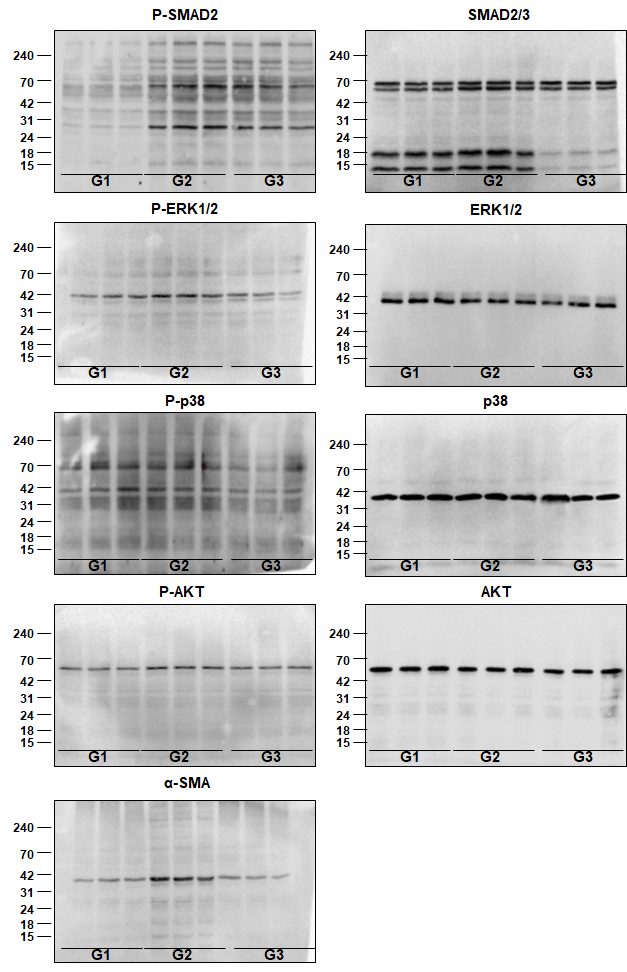


**Figure S2. Immunoblotting assay of p-SMAD2, SMAD2/3, p-ERK, ERK, p-P38, P38, p-AKT, AKT, α-SMA and β-actin protein expression in TGF-β-stimulated A549 cells.** A549 cells were incubated with serum-free media for 24 h, and then stimulated with the absence or presence of TGF-β1 (10 ng/mL) or Rb (30 nM) for 30 min or 72 h. G1 is the untreated group (*n*=3). G2 is treated with TGF-β1 (*n*=3). G3 is treated with TGF-β1 and radotinib (*n*=3). Y axis indicates molecular weight (kDa).


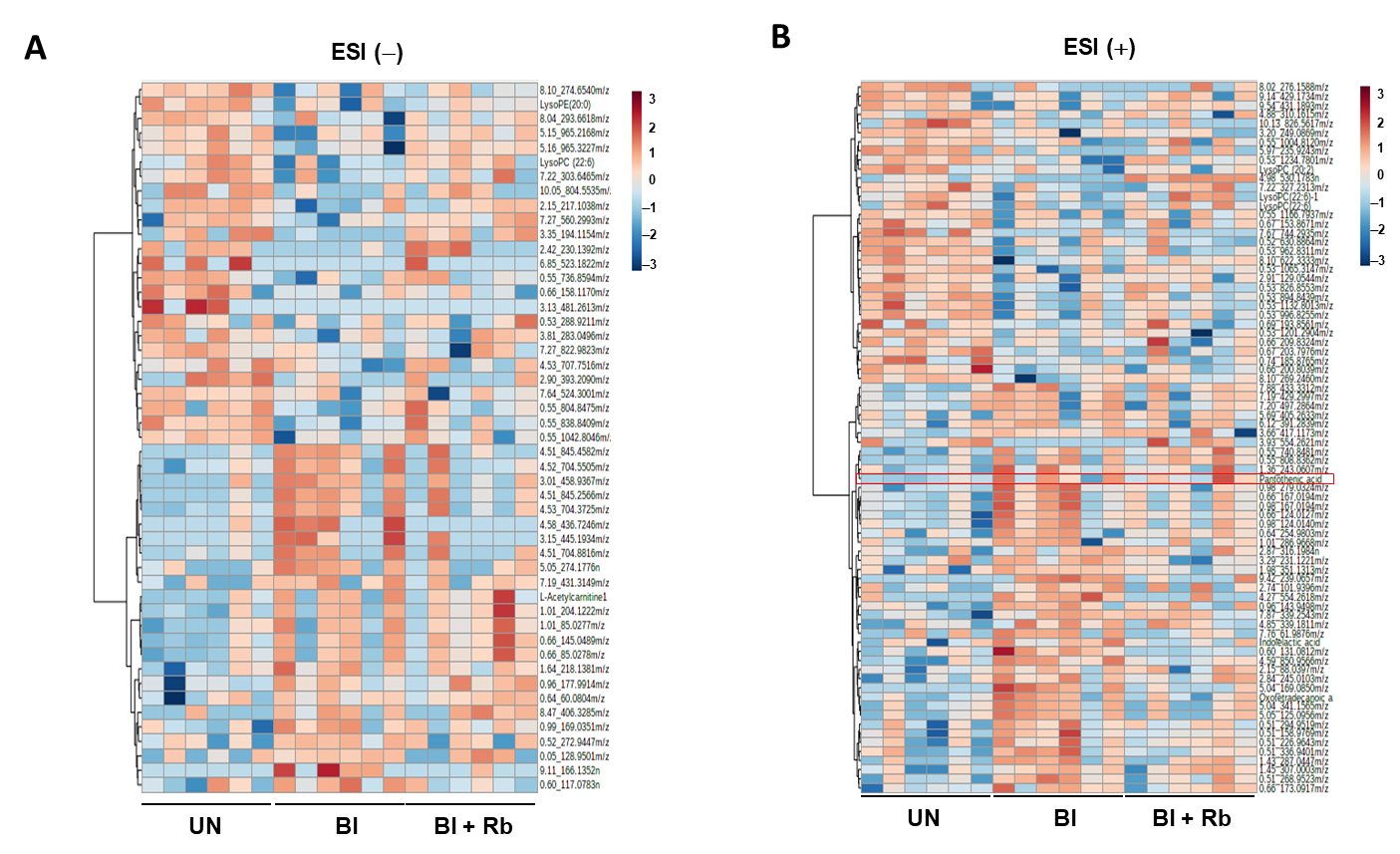


**Figure S3. Heatmap of metabolites ions differentially regulated among groups.** (A and B) Heatmap shows relative abundance patterns of metabolites ions (annotated and un-annotated) that has significantly different abundance between control and bleomycin groups (49 in ESI (+) mode and 78 in ESI (-) mode). Clustering within the heatmap shows a clear distinction of metabolite ions between control (UN; untreated group) and bleomycin (Bl: treated with 5 mg/kg of bleomycin /daily for 21 days) groups. Clustering within the heatmap also shows a distinction of several metabolites between Bl and bleomycin + radotinib (Bl+Rb: treated with 5 mg/kg of bleomycin and 30 mg/kg of radotinb /daily for 21 days) groups. The red box indicates the pantothenic acid. Bleomycin: Bl, Radotinib: Rb.


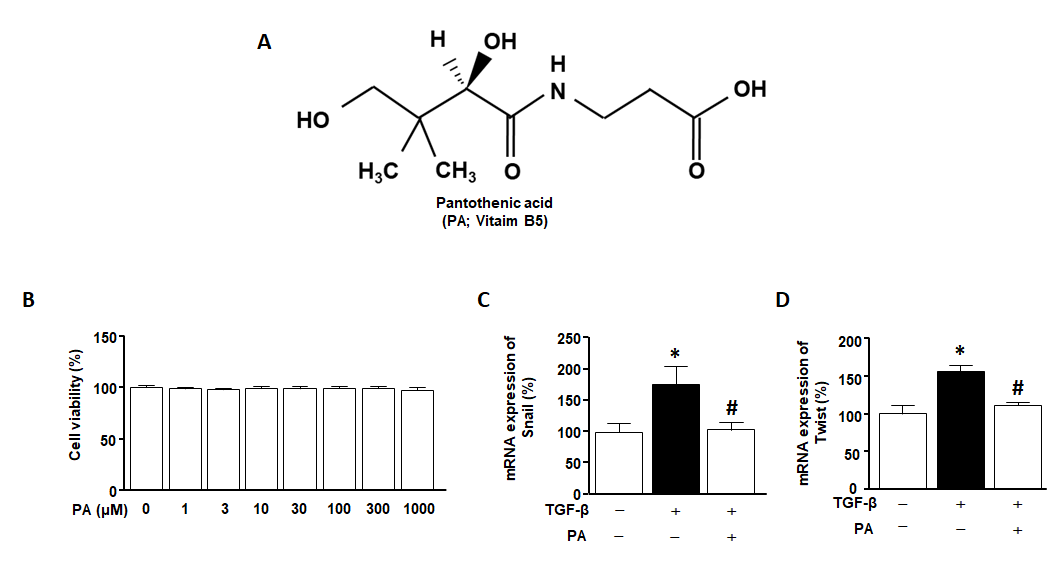


**Figure S4.** **The epithelial mesenchymal transition inhibitory effect of pantothenic acid on TGF-β-induced A549 cells.** (A) Chemical structure of pantothenic acid (PA). (B) A549 cells were incubated with serum-free media for 24 h, and then treated with PA (1, 3, 10, 30, 100, 300 and 1000 μM) for 48 h. Cytotoxicity was determined using the 2,3-bis[2-methyloxy-4-nitro-5-sulfophenyl]-2H-tetrazolium-5-carboxanilide assay. (C and D) The cells were incubated with or without TGF-β1 (10 ng/mL) and PA (30 μM) for 48 h. The mRNA expression of Snail and Twist were normalized to β-actin. The untreated group (UN) is expressed as 100%. Data are expressed as the means ± standard deviations (*n*=3). * *p* < 0.05 vs. UN; # *p* < 0.05 vs. TGF-β1.

Figure S4 shows following data:

PA has a chemical formula of C_9_H_17_NO_5_, and a molecular weight of 219.23 (Fig.S4A). PA showed no toxicity up to 1000 μM in A549 cells (Fig.S4B). In subsequent experiments, the optimal PA was set as 100 μM. A549 cells were incubated with or without TGF-β1 (10 ng/mL) and PA (100 μM) for 48 h. The mRNA expression of Snail and Twist was increased in the TGF-β1 group. In contrast, PA significantly reduced the TGF-β1-induced Snail and Twist mRNA expression (Fig.S4C and D).
